# Identifying Risk Factors for Shiga Toxin–producing *Escherichia coli* by Payment Information

**DOI:** 10.3201/eid1801.111044

**Published:** 2012-01

**Authors:** Hendrik Wilking, Udo Götsch, Helma Meier, Detlef Thiele, Mona Askar, Manuel Dehnert, Christina Frank, Angelika Fruth, Gérard Krause, Rita Prager, Klaus Stark, Boris Böddinghaus, Oswald Bellinger, René Gottschalk

**Affiliations:** Robert Koch Institute, Berlin, Germany (H. Wilking, M. Askar, M. Dehnert, C. Frank, G. Krause, K. Stark);; Health Protection Authority, Frankfurt am Main, Germany (U. Götsch, B. Böddinghaus, O. Bellinger, R. Gottschalk);; Veterinary Service, Frankfurt am Main (H. Meier, D. Thiele);; Robert Koch Institute, Wernigerode, Germany (A. Fruth, R. Prager)

**Keywords:** foodborne diseases, bacteria, Shiga-toxigenic, Escherichia coli, Shiga toxin–producing disease outbreaks, STEC, hemolytic-uremic syndrome, case–control studies, enteric infections

**To the Editor:** During May and June 2011, a large outbreak of hemolytic uremic syndrome (HUS) and diarrhea caused by Shiga toxin–producing *Escherichia coli* (STEC) occurred, centered on northern Germany (*1**,**2*). Early on, salads and raw vegetables were suspected to be food vehicles (*3*). Also in May, the staff department of a local company informed the Health Protection Authority in Frankfurt in southwestern Germany about the rapidly increasing number of patients with bloody diarrhea and HUS among employees at 2 company office sites. Both sites were served by cafeterias run by the same caterer. Main dishes were prepared in the cafeterias’ kitchens and differed between the 2 sites. However, in both cafeterias various fresh foods from a salad bar and fruits, desserts, and daily asparagus dishes originated from the caterer’s main kitchen. The salad bar included 30 items. Suspecting that this outbreak was linked to the one in northern Germany, we conducted an outbreak investigation to confirm the epidemiologic link to focus epidemiologic and traceback investigations.

A face-to-face survey among hospitalized employees and by email among all other employees was conducted, which included personal details, symptoms, and information about general food eaten at the cafeterias. We defined outbreak cases as infections in employees of the company at 1 of the 2 sites who by May 23, 2011, were either hospitalized with bloody diarrhea or HUS or who self-reported onset of bloody diarrhea from May 8 through May 23. A total of 320 persons responded to the survey, and 285 (89%) of 320 of the responders stated they used the cafeterias; 60 employees fulfilled our case definition. Case-patients’ median age was 33 years (range 22–60 years); 36 (60%) of 60 were female. Thirty case-patients were hospitalized; HUS developed in 18 (30%) (Figure A1). Disease onsets occurred over 9 days. Beginning and magnitude of the outbreak were not different between cafeteria locations. Bacteriologic diagnostics for 11 patients yielded results that are compatible with the outbreak strain (*4*).

We used billing data from the cafeterias’ obligatory cashless payment system to ascertain risk factors for disease. A nested case–control study design was chosen, limited to a fraction of the cohort to obtain rapid risk estimates. Exposures included were purchases of any fruit, salad bar item, dessert, or asparagus dish in either cafeteria from May 2 through May 13. On the basis of customer identification numbers, the caterer provided billing information for persons with early cases (n = 23). Controls were randomly chosen persons from the caterer’s database whose disease status was checked against the survey information (n = 30) and who did not report symptoms of diarrhea (nonbloody), vomiting, or nausea during the same period. Univariable logistic regression was performed.

In univariable analysis, salad bar purchases were highly associated with illness (odds ratio 5.19; 95% CI 1.28–21.03), and desserts, fruit, and asparagus dishes were not (Table). Three (9%) of the case-patients remained unexposed to salad bar items according to the payment system data. The analysis of main courses purchased in 1 cafeteria revealed that no such meal had been consumed by >5 (22%) of 23 case-patients. Beginning May 23, the cafeterias were closed for 1 week, and salad sales were suspended for a longer period. There were no additional cases.

**Table:** Univariable analysis of risk factors for bloody diarrhea among users of 2 cafeterias in Frankfurt, Germany, 2011

Risk factor	No. case-patients exposed/ total no. (%)	No. controls exposed/ total no. (%)	Univariable analysis*	
			Odds ratio (95% CI)	p value
Salad bar	20/23 (87)	16/30 (53)	5.83 (1.42–23.88)	0.014
Dessert	16/23 (70)	18/30 (60)	1.52 (0.48–4.81)	0.473
Fruits	5/23 (22)	10/30 (33)	0.53 (0.15–1.81)	0.312
Asparagus dish	7/23 (30)	11/30 (37)	0.76 (0.24–2.41)	0.635
Female sex	16/23 (70)	15/30 (50)	2.28 (0.73–7.15)	0.155
Age, y				
<30	12/23 (52)	6/30 (20)	2.80 (0.62–12.66)	0.181
30–<40	5/23 (22)	7/30 (23)	Reference	Reference
40–<50	4/23 (17)	13/30 (43)	0.43 (0.09–2.14)	0.303
>50	2/23 (9)	4/30 (13)	0.70 (0.09–5.43)	0.733

*Estimates in a multivariable model remained virtually unchanged.

These results and the identification of the same rare serotype of O104:H4 renders this a satellite outbreak to the larger outbreak in northern Germany, which is the largest outbreak in terms of HUS ever described worldwide. Sprouts are believed to be the food vehicle (*5*). Sprouts available in the Frankfurt cafeteria salad bars were traced back to a producer of fenugreek sprouts, which appear to be the common source of primary cases in the entire outbreak (*5*). Sprout consumption could not be studied directly in Frankfurt because of the intense media attention on the sprout hypothesis once it had been announced. Also, it was thought that too much time had passed to successfully recall actually selected salad bar items consumed a few weeks previous.

Cafeteria billing information allowed for a rapid investigation while avoiding exposure misclassification attributable to ill-remembered food purchases (*6*). Using data sources independent of individual memory is quite useful. In previous studies, similar tools were successfully applied for the detection of outbreak vehicles. Credit card information was used during an investigation on STEC in beef sausages in Denmark (*7*), supermarket purchase records for STEC in Iceland (*8*), and grocery store loyalty card records for cyclosporiasis in Canada (*9*). Shopper card information was used in the United States in an outbreak of *Salmonella enterica* serovar Montevideo (*10*). However, billing information also could have introduced exposure misclassification, e.g., purchased food that was left uneaten or brought for colleagues. Analysis on ingredient level is often not possible. This study emphasizes the need for recall-independent investigation methods. In settings where such methods are available, they should be exploited early and relevant data saved from routine deletion.

## Figures and Tables

**Figure A1:**
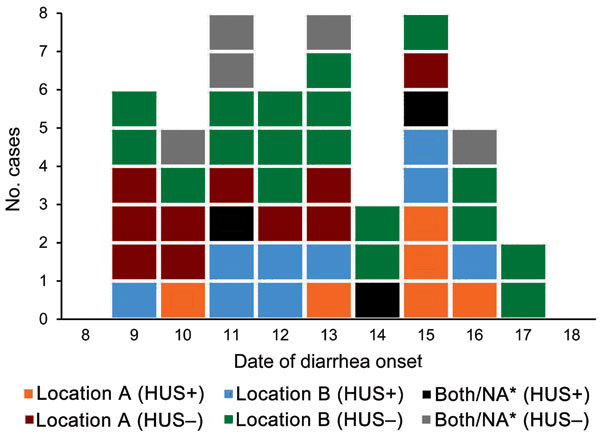
Patients with Shiga toxin–producing *Escherichia coli*/hemolytic uremic syndrome (STEC/HUS) by onset of diarrhea and cafeteria visit (location A or B) during STEC/HUS outbreak at a company in Frankfurt, Germany, 2011 (N = 51). NA, visited both cafeterias or specification was missing.
