# Correction: Vol. 18, No. 1

**DOI:** 10.3201/eid2508.C12508

**Published:** 2019-08

**Keywords:** errata, erratum, correction

An odds ratio and 95% CI were incorrect in Identifying Risk Factors for Shiga Toxin–producing *Escherichia coli* by Payment Information (H. Wilking et al.). The correct data for salad bar purchases were odds ratio 5.83, 95% CI 1.42–23.88. The article has been corrected online (https://wwwnc.cdc.gov/eid/article/18/1/11-1044_article).

